# “We don’t get asked”: including people with workforce experience as public contributors in a workforce-related study about homecare work towards the end-of-life

**DOI:** 10.1186/s40900-026-00931-3

**Published:** 2026-09-04

**Authors:** Helen Roberts, Samina Begum, Michelle Dale, Helene Elliott-Button, Justine Krygier, Colin Moss, Miriam J. Johnson, Liz Walker

**Affiliations:** 1 Institute for Clinical and Applied Health Research, University of Hull, Hull, HU6 7RX UK; 2 Involve Hull, University of Hull, Hull, HU6 7RX UK; 3 Wolfson Palliative Care Research Centre, Hull York Medical School, University of Hull, Hull, HU6 7RX UK; 4 Faculty of Health Sciences, University of Hull, Hull, HU6 7RX UK

**Keywords:** Public involvement, End-of-life care, Palliative care, Homecare workers

## Abstract

**Background:**

Homecare workers are an undervalued but essential part of the health and care workforce in England, providing vital end-of-life care without adequate training or support. Through the SUPPORTED study we explored the experiences, training and support needs of homecare workers who care for people with advanced illness, and co-developed (with homecare workers, managers, and educators) new training resources and recommendations to improve policy and practice.

**Main body:**

This paper explores the public involvement in this study and the contribution made to the research process, research outputs, relationships between the people involved, and our understanding and practice of public involvement. We involved 23 people with personal experience of this topic in different roles. We worked with five public contributors (homecare workers and family carers) during the study inception and grant application stage. Following successful funding, we set up two advisory groups (homecare workers; service users and family carers) which met regularly to support and inform the study from protocol development onwards, including sense-checking interview findings and training resource development. We also included lay people with topic experience in research team management meetings and study oversight. The distinctive features of our approach included creating parallel streams of public involvement; giving voice to the workforce being studied; involving lay people in multiple roles and levels of study governance; integrating public involvement with qualitative research and co-design methods; sustaining a flexible, listening and adaptive approach; and using creative methods to understand and evaluate our work.

**Conclusion:**

By using this approach, we successfully involved a workforce historically absent from research, with little influence on decision making in their working lives. Involving many people over a sustained period helped us learn from diverse perspectives and resulted in changes to our approach, for example, recruiting homecare workers directly instead of through managers, and better language to describe the study population. A dedicated coordinator role was critical in sustaining relationships and ensuring public voices were immersed into all aspects of the study. This had reciprocal benefits for public contributors, the research team, and the quality of the research, shaping our research and involvement practice and influencing the direction of the study.

## Abstract (de)

In this paper we provide a commentary on how we involved homecare workers, people receiving homecare and their unpaid carers in a research study about homecare work at end-of-life. The SUPPORTED study took place in three sites in England including two northern cities and one south London borough. We interviewed people to find out about the training and support needs of homecare workers who provide care to people with advanced illness. Based on what people told us, we then co-created (with homecare workers and managers) new training resources and recommendations to help people working in homecare and those who plan and fund services. We involved 23 people with personal experience of this topic in different roles in the study. Our approach was built around conversations, which began at an early stage. We set up two advisory groups to support and inform the study. One of these groups was set up to give a voice to the workforce being studied. We also included lay people in research team management meetings and in an oversight role. Working together in this way helped us to undertake the study, understand the data, co-design the training resources and other outputs, and share the findings, in a complex process of mutual learning. Our homecare worker advisors learned that their voice was important, that researchers wanted to listen to them and that what they said mattered. This was one of the ways in which we made an important difference to those we involved.

## Background

*Some will say I am just a carer*,* but I am the glue that holds it all together…* (Not Just a Carer narrated video, [1])

Social homecare workers (also known as home helps, aides, personal assistants etc.) are an undervalued but essential part of the health and care system in England and elsewhere with ageing populations [2]. Homecare workers care for people wishing to stay in their own homes at end-of-life, yet training and support for this workforce is often poor, inconsistent, or totally unavailable. Homecare workers are employed by private sector or council-run agencies and experience low pay, job insecurity, lack of career progression and isolated working conditions [3].

Little is known about the experiences of homecare workers or their training and support needs in providing care for people at end-of-life [2, 4]. We carried out the SUPPORTED study to address this gap [5]. In three diverse sites in England (see Table 1) we explored the experiences and training needs of homecare workers from different perspectives, including varied socio-economic and ethnic backgrounds [3, 5]. Our research activities are described in Table 2.

**Table 1:** Sites in the SUPPORTED study

Site 1	A northern city with a diverse ethnic population and significant levels of poverty, alongside pockets of affluence in the surrounding, more rural area
Site 2	A south London borough, which was relatively affluent, with an ageing population and relatively low ethnic diversity
Site 3	A northern city with high indices of multiple deprivation and a substantial majority of the population identified as White British

**Table 2:** Research activities in the SUPPORTED study

Interviews	Interviews with 133 key stakeholders (homecare workers and managers, health and social care staff, people receiving care, and their unpaid carers) across three sites [3]
Policy review	Documentary analysis of policy documents and existing training provision in end-of-life care for homecare workers [6]
Co-design workshops	Co-design with 77 homecare workers and managers of: • end-of-life care training resources [7] • recommendations for training and building communities of practice to support homecare workers providing end-of-life care

Our public involvement plan was ambitious, bringing in the voices of homecare workers, people receiving homecare, and their unpaid carers. In this paper, we report how we achieved this and reflect on what we learned about the benefits and challenges of sustained public involvement in an end-of-life care study, from research funding proposal development (July 2021) through to the dissemination of findings (June 2025). We present how our approach contributes to nuanced and critical understandings of public involvement [8, 9], and advocate for a more flexible, holistic and person-centred definition and practice of public involvement in research, particularly with regard to involving people with workforce experience where such people are study research subjects.

## Note about language

In this paper we avoid the use of ‘PPI’ and ‘patient and public involvement’ to describe our lay contributors; this was a social care study with people receiving and delivering care services. Rather, we use the terms ‘public contributors’, ‘contributors’, ‘advisory group members’, and ‘public members’ and describe their relationship to the provision of homecare at end-of-life. We use the term ‘public involvement’ to mean the inclusion of lay people as advisors in the research process, based on their experiential knowledge of the topic being studied.

In involving homecare workers, we follow the definition of public involvement used by the National Institute for Health and Care Research (NIHR) in England, which includes “people from organisations that represent people who use services” [10]. We accept that this should not be used as a way for healthcare professionals to speak *for or instead of* patients, to avoid powerful voices speaking for less powerful voices. Homecare workers are far from powerful within the workplace or within research. We make the case in this paper for the inclusion of homecare workers within the scope of public involvement, both as advocates for the people that they care for and as a workforce that is seldom heard by society and under-represented in research.

## Aim

*Invite many voices to the table; create multiple tables if that is what works for voices to be heard* [11]

Our aims for the public involvement in this study were:

1. To involve people with personal experience of receiving or providing homecare towards end-of-life, including those living with advanced illness, unpaid carers, and homecare workers.
2. To involve people with diverse perspectives, backgrounds, and experiences of homecare towards end-of-life.
3. To advise and inform our approach, understanding the data, co-designing the training resources and other outputs, and sharing the findings, through mutual learning.

The first aim reflects the importance of involving enough people with specific experiential knowledge of the research topic to gain a holistic view. This included representatives from the workforce being studied, which was unusual but essential to ensure that the principal beneficiaries of the research had a say in the research process.

## Our approach

*[Public involvement] is a time consuming*,* emotional*,* cognitive and practical effort* [12]

**Fig. 1:**
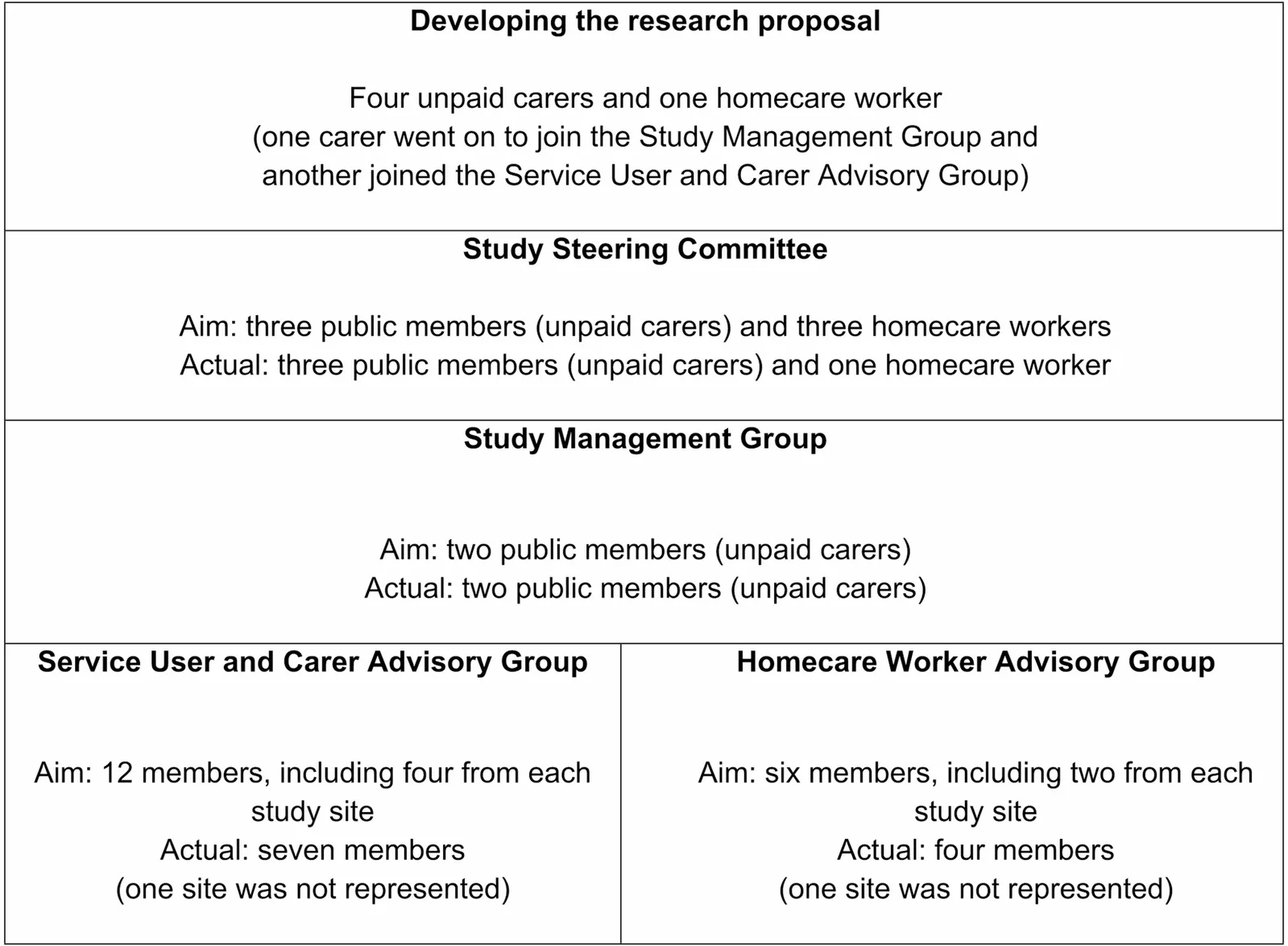
Public involvement in the study

Our approach included involving homecare worker representatives and not just those with experience of receiving care and involving lay people in advisory roles at all levels of the study (Fig. 1).

Practically and conceptually, we included homecare worker advisors within the scope of public involvement. Our rationale was that homecare workers needed a voice not just in sharing their experiences (through qualitative interviews) and co-designing the training resources (through workshops for workers and managers), but also in planning and carrying out the study – and this is what we understand public involvement to be.

Our plan for public involvement sought to weave in public voices at multiple levels of the study in different roles, demonstrating what could be learned from an ambitious approach. Our research funder NIHR requires there to be at least one public member on a study steering committee and encourages the inclusion of public co-investigators on research teams [13, 14]. The decision to create a separate homecare worker advisory group was designed to deal with power dynamics among public contributors [15]. In a small group confidence to speak up could develop among people who had no prior experience of involvement in academic research and ensure that they were heard without being overshadowed by more experienced public contributors with a carer or service user perspective. Thus we created the ‘multiple tables’ of public involvement recommended by Plamondon [11].

Within this study we developed a collaborative and conversational way of working with public contributors and our use of ‘we’ in this paper reflects this. This does not mean that we were always in agreement, or that there were no power imbalances present. However it does mean that we strove to set up a governance framework that enabled us to listen attentively to every voice and where we could not achieve consensus, to listen to dissent before proposing a way forward [16, 17].

We brought public involvement together with qualitative research and co-design methods to gain a holistic perspective [18]. Reflecting this, three of our public contributors are co-authors on this paper (CM and SB (both members of the study management group), and MD (member of the homecare worker advisory group)). Their contributions included reading and commenting on successive versions of the manuscript so that their views and experiences were strongly represented in the final version.

### Developing the research proposal

Public involvement began before the research grant was awarded. University of Hull funding enabled discussions with five public contributors about the relevance and value of the research, how to ensure diversity amongst participants, and the choice of language to describe homecare and end-of-life. This improved the quality of our application, and our plans for public involvement. For example, we stopped using the terms ‘social carers’ or ‘domiciliary care assistants’ in our research proposal and adopted the term ‘homecare workers’ which is in more common usage, as well as replacing ‘patient and public involvement’ with ‘service user and carer involvement’, to make clear that this was a social care study. Most importantly, it was from these early conversations that we first saw the need to involve homecare workers in an advisory role alongside those with experience of receiving homecare.

### Leadership and coordination

Public involvement was led by a full-time public involvement coordinator (HR) working across research studies within the Institute for Clinical and Applied Health Research at the University of Hull. HR was costed as a grant co-applicant, and played a central role in the research team including study management group membership. One co-chief investigator (LW) took the academic lead for public involvement, ensuring support for HR and prioritisation of public involvement activities by the whole team.

### Setting up the advisory groups

HR set up two public advisory groups, meeting online two to three times a year. One included those living with advanced illness and unpaid carers, and the other, homecare workers (see Fig. 1), ensuring dedicated spaces for each.

To reach homecare workers, we initially advertised on social media. Forty-seven people responded. During screening (meeting individually online and using a screening questionnaire), we found many were not genuine - unwilling to use their camera; using the same phrases to describe end-of-life care; and giving unconvincing answers about where they worked.

We therefore excluded social media contacts and successfully advertised the role to members of Involve Hull [19], the University of Hull’s public involvement network for health and care research. The group initially had no representation from sites one and two, but membership was refreshed during the study as necessary (see Table 3 for membership summary), which increased the geographical spread. We collect demographic data as part of recruitment to Involve Hull and followed the same approach in this study.

**Table 3:** Membership of homecare worker advisory group

Gender	2 men 3 women
Ethnicity	2 White British 3 Black African
Location	1 from site 1 3 from site 3 1 from another area (no members from site 2)
Age range	30–60 years
Employment status	4 in employment 1 self-employed due to health status
Homecare worker role	1 with current experience as a care coordinator 4 with current or past experience as care workers 1 undergoing training
**Total people involved**	**5**

We sought to reach unpaid carers and people living with advanced illness through Involve Hull, our academic partners’ public involvement networks and contacts with local carers’ organisations. We also placed a general call for people with personal experience of palliative care on the national People in Research website [20]. The service user and carer advisory group originally had six members, with one more person joining midway. Some members withdrew for health, work and personal reasons whilst others stayed for several years giving continuity (see Table 4).

**Table 4:** Membership of service user and carer advisory group

Gender	2 men 5 women
Ethnicity	4 White British 3 Asian British
Health status	1 person with personal experience of living with advanced illness 3 people living with a long-term health condition
Caring status	6 people with personal experience as unpaid carers
Location	3 people from site 2 3 from site 3 1 from another area (no members from site 1)
Age range	25–80 years
Employment status	3 retired 3 in employment 1 not working due to caring role
**Total people involved**	**7**

Alongside group meetings, members could complete activities in their own time, contribute by email and in one-to-one conversations with HR or research colleagues.

### Running the advisory groups

We cultivated welcoming places and relationships [11]. Following individual conversations, we held welcome meetings to introduce the study, the research team and each other before our work began. This was repeated when new members joined. Members of the research team attended meetings in small numbers providing a balance of researchers and group members. Meetings were hosted by HR and arranged to suit group members. All materials were shared in advance in good time to allow preparation. We wrote in Plain English and discussed our work without jargon to ensure maximum participation and engagement.

**Fig. 2:**
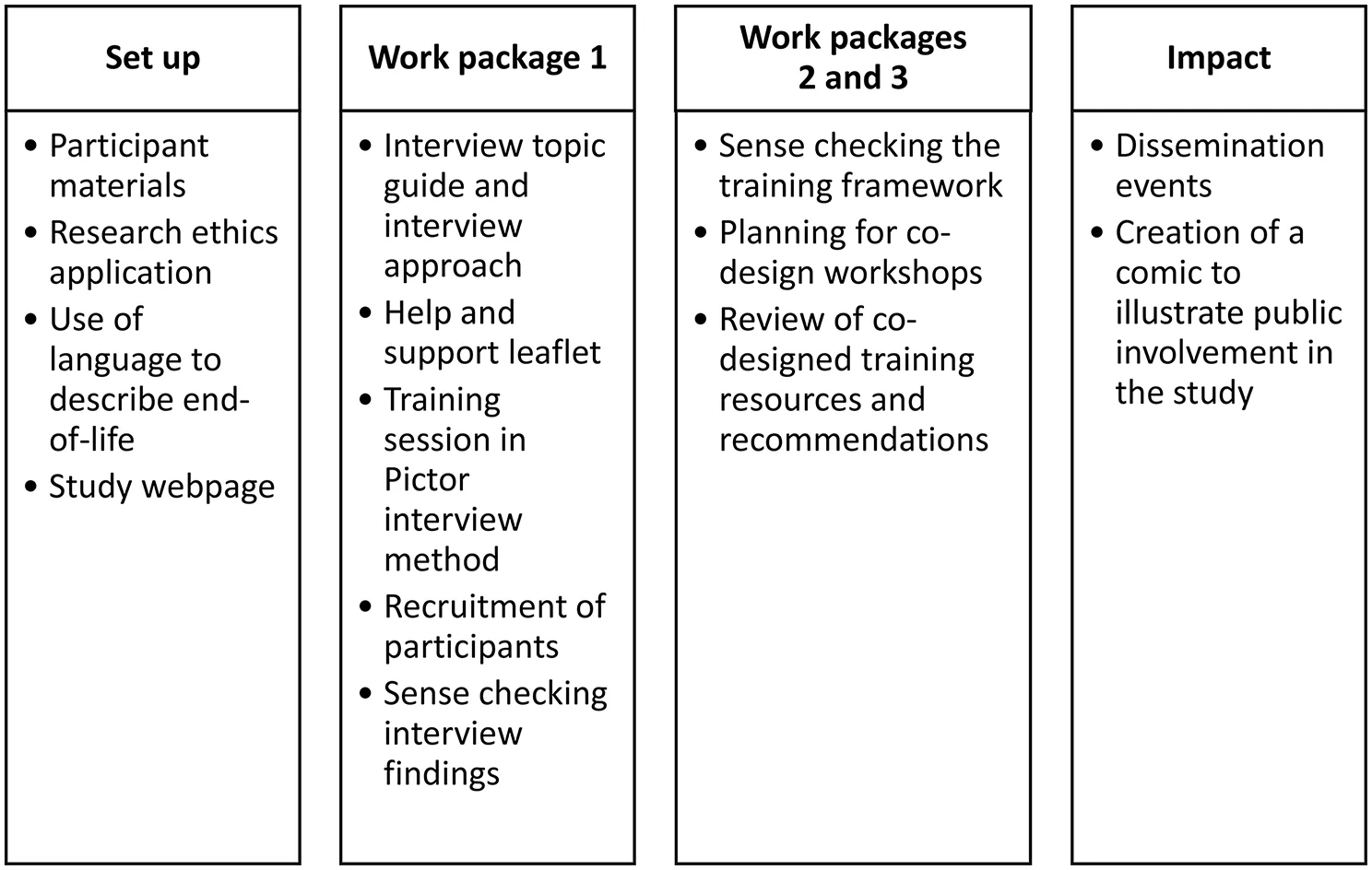
What did we involve our advisory groups in?

We involved both groups in the same activities and conversations in parallel. Figure 2 shows how the groups fed into different stages of the research.

### Including public representatives on study oversight groups

CM and SB represented unpaid carers on the study management group. Public involvement was the first standing agenda item, and both had an open invitation to comment and ask questions on any topic. Having two public members, supported by HR, gave more weight to their voice. They were included in group email correspondence and given access to the study document repository, including selected anonymised interview transcripts. As the study progressed, they co-facilitated service user and carer advisory group meetings. We also involved them in some work package meetings giving greater involvement and insight into areas that interested them. This immersion into all aspects of the study enabled fuller participation with reciprocal benefits to the public members, the research team, and the quality of the research.

Our steering committee was well-balanced between academic, practitioner and lay members. Three people had experience as unpaid carers and one homecare worker was recruited through their trade union. Table 5 summarises the public membership of oversight groups.

**Table 5:** Public members on oversight groups

Gender	1 man 5 women
Ethnicity	4 White British 2 Asian British
Health status	2 people living with a long-term health condition
Caring status	5 people with personal experience as unpaid carers
Location	2 people from site 1 2 people from site 3 2 people from other areas (no members from site 2)
Age range	30–80 years
Employment status	2 retired 3 in employment or self-employment 1 not working due to caring role
**Total people involved**	**6**

### Support and learning

HR offered small group or one-to-one conversations to help members prepare for meetings and provide support. Personal experiences were sometimes revisited within meetings. Some were distressed at hearing others’ experiences or felt uncomfortable expressing personal feelings alongside work related issues. We mitigated unresolved distress by developing open, supportive and responsive relationships, mainly through HR [21].

We organised online learning events and created learning guides on topics including the lifecycle of a research study; public involvement roles and groups; research ethics; data protection and confidentiality; and diversity in research. Two members spoke about their experiences at learning events, including an international roundtable (UK, Australia) about public involvement / consumer engagement [22].

We offered NIHR-recommended payment for time spent in meetings and other research-related expenses [23], with higher payments for oversight roles recognising the greater responsibility and preparatory work.

### Capturing activities and feedback

We maintained a record of public involvement contributions and their impact to encourage constant evaluation, responsiveness, and inclusion (see Table 6). Towards the end of the study, we surveyed our public contributors (completed by 11/16 people) and held an online feedback session for the homecare worker advisory group.

**Table 6:** Ways of capturing activities and impact

Method	Frequency
Activity and impact log	Continuous, from September 2022 to June 2025
Regular progress reports to the research funder	Every six months
Notes of advisory group meetings, one-to-one and small group conversations	At least every six months from October 2022 to May 2025
‘You said, we did’ table summarising the review of participant information prior to research ethics submission	November 2022
Feedback surveys for public contributors	December 2022, January 2024 and February 2025
Feedback survey for researchers	December 2023
Reflective online feedback session for homecare worker advisory group	March 2025
Online workshop with graphic artist for public contributors	May 2025
Comic story (available online and as poster and booklet versions)	May to October 2025

We commissioned a graphic artist to work with our public contributors to create a comic about their involvement, holding a workshop in which people responded to initial drawings with ideas about the artistic approach, metaphors for our work, the characters in the story, representing homecare at end-of-life, and aspects of their experience [24].

## The difference we made

*Involvement is evolutionary*,* in unpredictably progressing through a series of interrelated episodes of learning* [9]

The two advisory groups had different strengths and weaknesses in terms of their contribution to the research ethics process, recruitment of participants, co-design process, and sense checking the interview findings and the training resources.

Our involvement of people from an early stage and throughout the study enabled strong relationships and understanding to develop. However the disadvantage of early involvement is that setting up research projects takes time, and can lead to frustration among public contributors - “you speak about the methodology in huge detail but what is the goal…?” (public contributor feedback survey, December 2022). Our public contributors’ focus was on policy and practice impact. We found a tension between the researchers’ methodological rigour, and public contributors’ desire to see practice improvements, as soon as possible. The learning events HR developed in response helped to address concerns and increase understanding.

### Contribution to the research ethics process

Before seeking ethical approval, the advisory groups reviewed participant-facing information materials for clarity and reading accessibility. They also reviewed our interview approach and questions, specifically the use of a graphic elicitation technique (Pictor) [25] alongside conventional interviews, to ensure acceptability and accessibility. This technique involves participants making a diagrammatic representation of care they have provided or received, and of their support network, and then ‘telling the story’ behind their diagram.

The service user and carer advisory group agreed with our stepped approach to information sharing to avoid overwhelming participants with information. Members felt that the Pictor technique would be unfamiliar, so we created a video and added a link in the participant information. They agreed with signposting participants to support information after their interview to mitigate potential distress, except for bereavement support information as participants might be unaware that death was close. The group felt that the interview questions were appropriate and acceptable for such a sensitive topic.

However, our experience of involving the homecare worker advisory group in this process was challenging. One member disengaged from the study after receiving the documents to review. Another found it difficult to see what feedback they could offer, instead completing the interview questions as if they were a participant. We had assumed that commenting on written documents was a straightforward activity; it was not. Our learning from this included not expecting everyone to take part in every activity, so that people could contribute according to their strengths, and ensuring that in future, interview topic guides are discussed through group conversation instead of being reviewed by email.

One homecare worker in the group identified that the consent form was potentially discouraging due to the way we described what would happen if poor practice or safeguarding issues were disclosed (a standard requirement by the Health Research Authority); we revised the wording to address this.

Advisory group input was described in the ethics application which reassured the ethics committee [26]. Before issuing approval, the committee questioned the best way to describe this study given some might not be aware that death was near. On the advice of our advisory groups, we altered our language to refer to ‘people living with advanced illness’ instead of ‘seriously ill’ or ‘nearing end-of-life’.

### Contribution to training our researchers

Three advisory group members took part in a training session for researchers about how to implement the Pictor method and were each paired with a researcher to practice this [25]. This was valuable for the researchers as many were new to this technique, but there were contrasting reactions from public contributors; one member found the training therapeutic in processing their own experiences, whilst another found it complex and was uncomfortable during the session. HR provided reassurance, avoiding a breakdown in relationships, but we decided not to repeat this approach when we ran the training a second time, as the focus was too academic to be accessible to all.

### Contribution to recruitment of participants

Recruitment initially proved challenging, particularly with a workforce unused to participating in research [27]. Both advisory groups gave pivotal advice, helping us to achieve our recruitment targets and a balance across the different participant groups and study sites. Our homecare worker advisors encouraged us to reach out to homecare workers directly rather than through their employers. They suggested creating short, accessible social media posts advertising the study. We took this idea to the steering committee and one member created a short animation for homecare workers for this purpose. Our service user and carer advisory group encouraged us to persist in recruiting participants in a particular study site to give greater diversity in our sample and gave advice about reaching participants from under-represented groups, for example South Asian communities.

### Contribution to sense checking interview findings

The researchers presented early findings to the advisory groups for sense checking and repeated this following interview completion. During this process, members reflected on their own experiences of receiving or delivering homecare at end-of-life, including in a special issue of our newsletter. This confirmed that the key themes made sense to those ‘on the ground’.

CM and SB requested to help with interview transcript coding, but this had not been planned for in the study protocol. Provision had not been made to train them for this role and it was essential that the same set of people were involved in analysing all 133 transcripts for consistency. As their involvement was not possible without affecting the rigour of the analysis, we came to a compromise. They were given controlled access to a sample of anonymised coded transcripts, alongside an explanation of the process and a copy of the coding framework. This gave them insight into the richness of the interview data and the painstaking work of data analysis, whilst also meeting ethics requirements. By being open about what we could and could not do in response to their request, and working out a way forward, we reassured them about the quality of the findings and sustained their engagement.

### Contribution to the co-design process

An initial homecare worker training framework was shared with the homecare worker advisory group for comment and their advice sought about the co-design workshops for homecare workers and managers. They recommended separate workshops for homecare workers and managers online and in-person to enable workers to speak freely and maximise access. Workshops avoided shift handovers and peak working, and dates were fixed a month ahead. This helped us reach more workshop participants, but we still encountered issues with engagement. The advisory group helped us to see that homecare workers were unused to working in this way; not normally consulted at work and not normally involved in developing training/educational materials.

### Contribution to sense checking the training resources

Early versions of our co-designed training resources and recommendations for education and training were shared with the advisory groups, as well as CM and SB, as part of a reviewing and refining process.

This helped the team to make important decisions about what the resources should look like and how they might work. For example, public contributors advised that the training videos be narrated by a voice that sounded approachable, clear, and understandable by homecare workers whose first language was not English. Therefore, we used a mixture of professional voiceover artists or AI-generated voices according to resources. Members shared ideas about how the training resources might be used in practice and different formats to support them. This informed our comprehensive set of ‘What if…’ cards as a resource for homecare workers and managers to use in training sessions, as a refresher, and an aid to decision-making in care situations. Their advice also informed how we approached specific training topics, including responding to the cultural and religious needs of people at end-of-life; what to do in the event of finding that someone had died or was dying; and language differences between those receiving care, their families, and homecare workers. In all these cases, their input helped us to improve the tone and language used in the resource, along with the detail of the content.

### Contribution to study management

CM and SB were unsure of their role initially and experienced some challenges. There was a disconnect between their personal knowledge of the research topic, and the purpose of the meetings in managing academic processes. The meetings were focused and timebound, experienced as pressure to be succinct and constructive. The research team, including the co-chief investigators (LW and MJ) who chaired the meetings, were respectful and inclusive, inviting and welcoming their contributions, and explaining acronyms and research jargon. However, CM and SB had difficulty judging when/if to contribute and sometimes felt ‘on the spot’ or overwhelmed when the intention of the research team was to be inclusive.

To mitigate against this HR held regular conversations with the public members to help them prepare for meetings and reflect on concerns [21]. Sometimes we could resolve an issue through making a practical change. For example, both found it technically difficult to access the project’s MS Teams site so instead we shared specific documents by email. Sometimes it was not possible to resolve an issue and we had to sustain an open dialogue instead. This was important means of support, as was having two public members – “the peer support was valuable and helped me grow in confidence, when at the start of the research I wasn’t sure where we were going” (public contributor feedback survey, February 2025).

Adding to the uncertainty about their role was a lack of any link between CM and SB and the advisory groups, preventing them from having a full picture of public involvement. We therefore invited them to service user and carer advisory group meetings, initially as observers, and then as co-facilitators. This benefited their understanding and engagement, and the quality of conversation within the advisory group.

### Contribution to study oversight

The importance of maintaining dialogue outside of formal meetings came out strongly in the feedback from public members of the steering committee. The opportunity to meet separately with HR prior to committee meetings enabled members to be better prepared, more engaged and more confident. One member highlighted the challenges of the role: ‘it can feel quite intimidating to be in a meeting with experts in the field’, and especially when ‘the time between meetings creates a distance from the research work’ (public contributor feedback survey, February 2025). A good relationship with HR, efficient administrative support, respectful and considerate chairing of the committee and a regular and informative newsletter were all mentioned as important to support them in getting the most from the role.

### Contribution to promotion and dissemination

We created a study webpage enlivened by public contributors’ and practitioners’ quotes [28], illustrated with appropriate images chosen with input from the homecare worker advisors. In between meetings we produced email newsletters to keep in touch and give space for members to share reflections and insights with their peers and the research team. Newsletters were added to the study webpage to communicate the progress of the research and show the value placed on the voices of public contributors.

Towards the end of the study, we organised events at hospices in our study sites to share findings and training resources with homecare workers and managers and other health and care practitioners. CM and SB helped plan a display about the public involvement, and attended their local event, alongside other public contributors. This gave them the opportunity to see the results of our work and talk to attendees about their role.

**Fig. 3:**
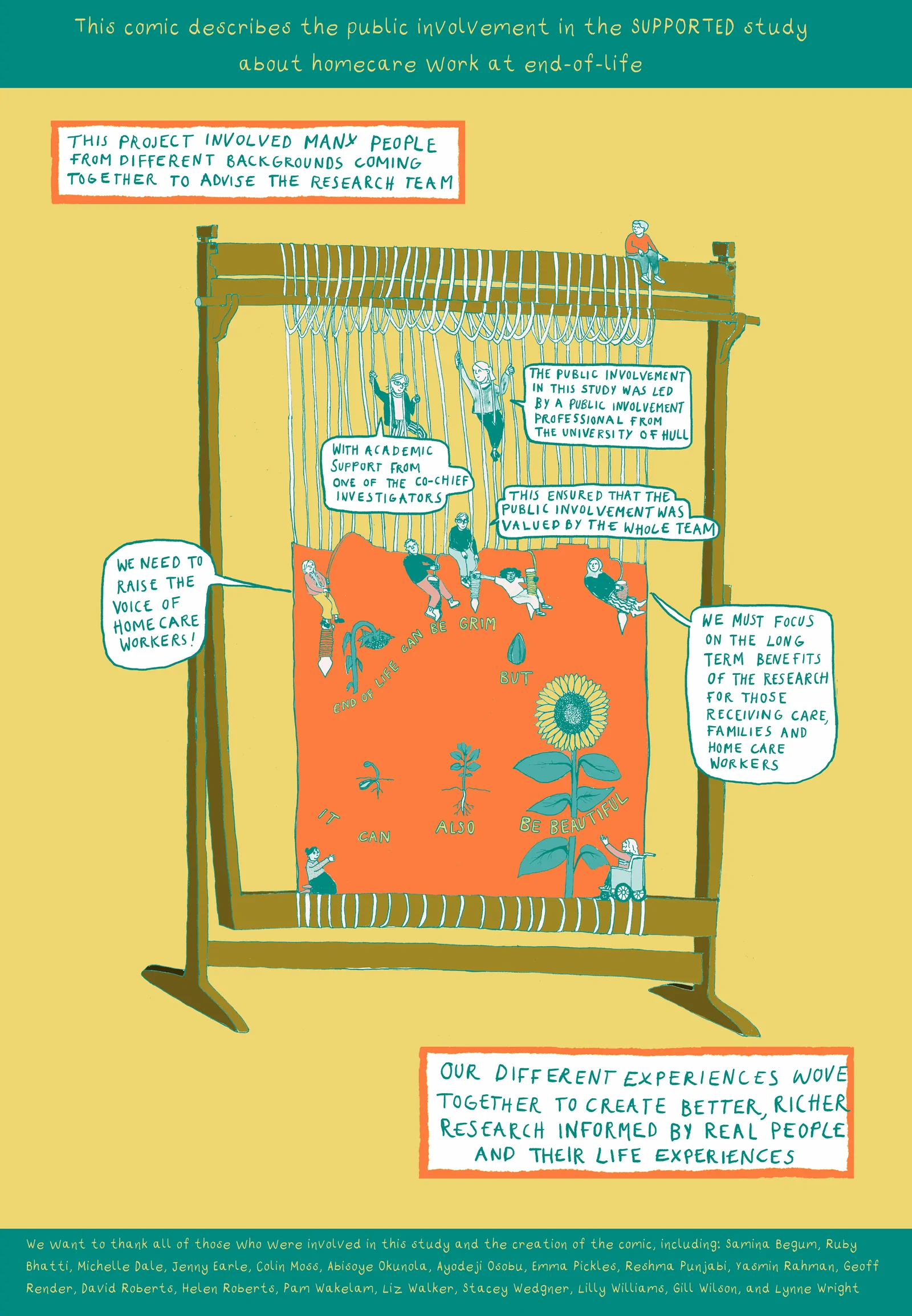
Image of tapestry on a loom representing public involvement in SUPPORTED (copyright Lilly Williams)

Our comic story used the metaphor of weaving to represent our collaboration. It is made up of two parts; an image of a tapestry of a sunflower being woven on a loom (see Fig. 3), and a panel-by-panel story of the involvement process. There was a strong sense that end-of-life can be grim, but it can also be beautiful.

The SUPPORTED team continues to share study findings and the training resources widely, influencing policymakers and encouraging uptake of the resources in practice. This included a visit to the Houses of Parliament as part of a showcase of work from the Wolfson Palliative Care Research Centre at the University of Hull. Two of our public contributors joined senior academics and spoke at this event and our comic was displayed.

### Working relationships

The quality of working relationships and engagement with the advisory groups was affected by several issues. HR provided reassurance and support for researchers and helped to build their confidence in undertaking public involvement, by mediating the relationship between them and group members [29].

Technical issues, lack of previous experience or a realisation that it was not what they expected or wanted to do, meant that some members disengaged or withdrew altogether. For others, it was due to declining health, or work commitments. A few did not put their cameras on during meetings and spoke very little. This was perceived negatively by other members of the group and affected the quality of conversations.

Group dynamics and levels of engagement could shift dramatically when a new person joined who was enthusiastic, happy to take part, and good at encouraging others to contribute. Offering payment for contributing in alternative ways outside of group meetings and adapting our approach to help people prepare better for meetings was beneficial. However, we struggled to resolve the tension between wanting to be inclusive and accommodating different needs.

### Homecare worker experiences of involvement

When we asked our homecare worker advisors to reflect on their involvement towards the end of the project, they felt strongly that they were respected by the research team: “I felt very well involved and I felt my views were being considered and there was mutual respect in the group as well” (homecare worker advisory group feedback meeting, March 2025). The group were new to public involvement and expressed trepidation about talking with academics. Some members left school without formal qualifications. Others had university level education, but English was their second language. Members were working alongside their involvement in the study and had busy lives; we had to fit around their commitments: “We’ve all got different working patterns, so yeah, there was some flexibility…they were kind of mindful of your time…and they got back to you in a timely manner’ (homecare worker advisory group feedback meeting, March 2025).

After early changes in membership, the group settled and developed into a supportive space that enabled members to contribute: “the way it was structured, everything was really good for my needs as well to take part. …And I think we bounced off really well on one another” (homecare worker advisory group feedback meeting, March 2025).

One person had experience as a homecare worker for young people with learning disabilities, of receiving homecare for a parent towards end-of-life, and of arranging homecare for themselves and described themselves as ‘flipping’ from one perspective to another and feeling ‘conflicted’ about their status in the group (homecare worker advisory group feedback meeting, March 2025). Another had experience of working in a hospice, as well as caring for a dying relative. We encouraged people to reflect from whichever perspective seemed most useful and did not stop someone in the homecare worker group advising from a personal point of view, or the other way round. However this remained a complex area for some members to navigate.

One of our homecare worker advisors found involvement in the study validated their experiences and gave them the confidence to request additional training from their employer. The emotional side of their involvement was cathartic [21] and this came through strongly in their feedback: “…this was my first project that I’d done and this has led on to others…this is the one I’ve enjoyed the most, right…when I sat back and thought about it, I thought we don’t get asked this question. We don’t get asked” (homecare worker advisory group feedback meeting, March 2025). As trust developed between members of the homecare worker advisory group, HR and the co-chief investigator (LW), this became a safe environment in which emotions and disagreements could be shared [30]: “And we weren’t just a number…we were people…I had a voice and what I had to say…was important” (homecare worker advisory group feedback meeting, March 2025).

## Discussion

*The way we see things is affected by what we know or what we believe… We only see what we look at. To look is an act of choice.* [31]

### Different ways of knowing

We chose to ‘see’ homecare workers, bringing different ways of knowing into the research process. People representing a health and social care workforce are often excluded from public involvement and viewed as having a ‘professional’ rather than a ‘public’ perspective [32]. We focused on the purpose and benefits of involving homecare workers, asking ‘What will they bring to the study that we would not otherwise know?’ [9]. Where a workforce group is being studied, including them in an advisory role alongside more traditional public involvement is invaluable, especially where that group is under-researched, rarely heard and has little influence on decision making in their working lives.

Among those we involved, some ‘wore many different hats’ [32]. Although our advisory groups tapped into distinct and different experiences, some members had experience from multiple perspectives. This proved to be insightful and reflective of the complexity of real life, showing that different stakeholder interests often combine within individuals and cannot be easily untangled [33]. There was still a value in distinguishing between different lay perspectives and roles in our governance framework because this gave a platform to the least powerful and enabled all voices to be heard in an equitable way. Public involvement groups must be managed flexibly and used as a *starting point*, enabling important conversations to develop within and outside formal meetings, rather than being used to contain involvement [9]. Our approach contrasts with those that only involve people in reviewing public facing study materials ahead of a research ethics application or only bring people into a study towards the end to support dissemination to public audiences [30].

### Documenting diversity

There is an evidence gap relating to the demographic profile of public contributors in research studies, due to the lack of routine systems to collect and analyse such data [34]. Statements about diversity in public involvement or the lack of it are frequently not supported by evidence [15]. National surveys [35, 36] need to be supplemented with ongoing data collection by established public involvement teams and within research studies. Without this we cannot plan for and achieve the diversity that we seek, nor understand where the gaps lie and address them [37].

### Visualising public involvement

Public involvement sits in an ambiguous space between ‘system’ (research) and ‘lifeworld’ (everyday life), where aims and motivations can conflict [38, 39]. Our approach reflects the value of sustaining a ‘space to talk’ [40], a continuous conversation throughout the different stages and across different sets of people to bridge the gap between us, in a process of ‘communicative action’ [41].

Combining public involvement, qualitative interviews and co-design methods enabled the research team to learn how these approaches can inform each other and improve the process of knowledge generation. Reflecting on our work, we found that we had used qualitative research practices within our public involvement activity without labelling them as such. This included methods such as member checking of early findings, dense description of the public involvement methods and demographic profile of our public contributors, and constant comparative analysis to repeatedly redevelop and refine our understanding in conversation with our public contributors [42]. This illustrates the ‘blurred boundaries’ between public involvement and qualitative research, and how the same practice can be given different labels [18]. Understanding this interplay of methods validates the practice of public involvement and highlights how it adds to the rigour of a research study.

Public involvement is a weaving together of different threads in the process of creating a shared endeavour (see Fig. 3). The tensions involved are normal, need care and attention, and are ultimately productive, creating a ‘space to change’ within a research study [40].

### *Not just* a public involvement coordinator

It was helpful to have HR whose specific team role was to coordinate the public involvement, acting as a ‘translation service’ between researchers and public contributors; something not always present or visible within a research team [9, 43]. Although HR was employed by a university, therefore not completely neutral, she sat outside the academic hierarchy, which enabled a conversational approach and creation of a welcoming space in the advisory groups. Direct support for HR from one of the co-chief investigators (LW) influenced the research culture within the team, illustrating that public involvement is part of ‘being a good academic’ [29].

Public involvement in research is hard. We ask contributors to share personal experiences, but not in too much detail, and reflect on what that might mean for something as technical as an academic research project. They do this within ‘a process designed for researchers, with knowledge that often contains emotions or feelings’ [21]. Public involvement has a paradox - we place people whose expertise and knowledge are personal and emotional into ‘factual spaces’ where emotions are not always welcomed [21].

The work involved in mitigating distress and caring for contributors is also hard. These skills are not ‘soft’ [43]. A strong theme from our research findings was that homecare workers saw themselves as ‘just a carer’, when in fact they juggled many skilled and complex elements in their role, building relationships with those receiving their care and their families. This is mirrored in a public involvement co-ordinator role; relationship building over time, both within and across research projects is work of care and cannot be reduced to a ‘deliverable’ without losing something important [44].

### More than a passive presence

Oversight roles remained the most difficult for public contributors. Reflecting on Crocker’s typology of public involvement roles, the public members of the study management group found it hardest to see the value in being a ‘passive presence’ [45]. However, they had significant impact on the researchers simply by being present at meetings, influencing the way researchers spoke and behaved. However, there were few opportunities for our public members to be ‘creative outsiders’ and ‘experts in lived experience’ due to the nature and purpose of the group (the place where the study bureaucracy happened). Meeting agendas were driven by research delivery within funding, governance and time constraints. Within this type of meeting, public contributors can feel like ‘limpets on the ship of research’ – “you are just stuck on the side of something, and people are very polite and they ask your opinion but basically they are doing what they want” [46]. However, CM and SB felt this to be an overly passive metaphor because over time they did contribute actively, whilst recognising that public involvement was not the focus of the management meeting.

Public involvement easily becomes a ‘tick box’ exercise, especially if its purpose is unclear. The UK Standards for Public Involvement [47] imply ‘the more public involvement the better’ [48], but our experience suggests this is not automatic. We adapted our approach to create a more fulfilling role for CM and SB outside of management group meetings [21] and addressed their expectations of what was possible within their role - but we did not always come to agreement [39].

Examples of public involvement challenges are important to share, as learning for others and to illustrate the complexity of the task [49, 50]. Some contributed very little, perhaps overstating their experience of homecare at end-of-life, mirroring our experiences of social media responses during the recruitment process [51–53]. These tensions can be mitigated but not designed away completely [49].

### Weighing the costs and the benefits

The approach taken in this study required a considerable investment of resources, time and effort. This was made possible by budgeting effectively for involvement activities in the research grant application and building on the existing infrastructure for public involvement within our research institute (including a well-established and diverse involvement network, supported by an existing programme of learning events and resources). This study benefited from our expertise and experience in this field, an institutional commitment to public involvement as a professional role, and the leadership of academics prepared to learn from the practice of involvement. Our experience shows that public involvement requires meaningful investment at a research funder and institutional level to allow meaningful practice.

**Fig. 4:**
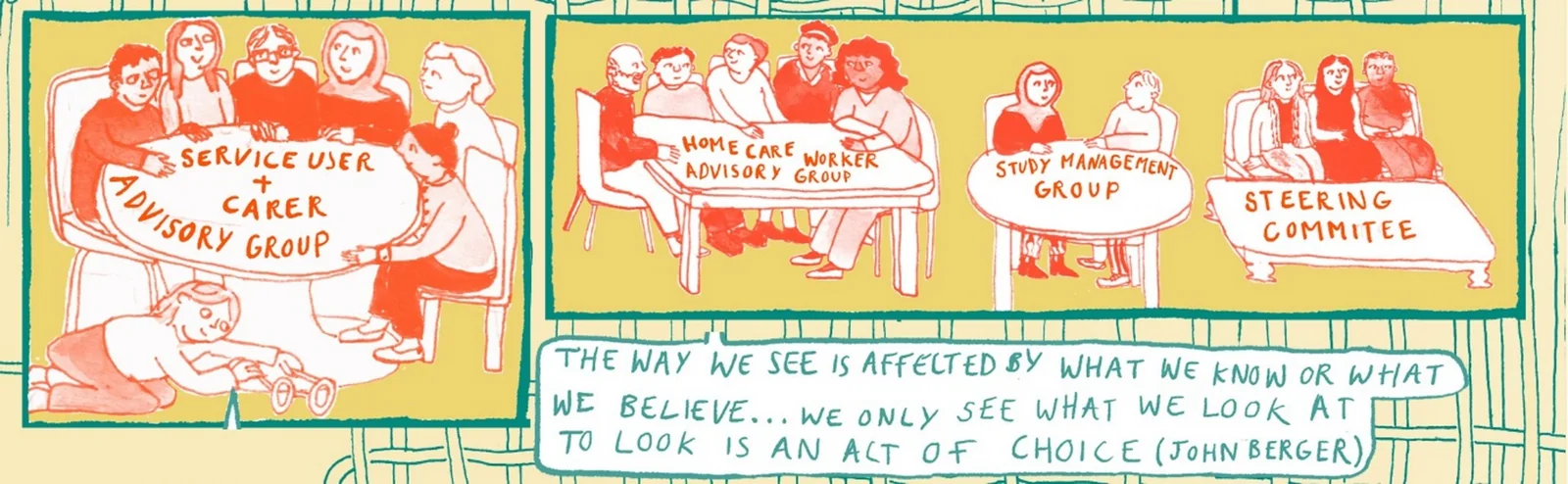
Excerpt from SUPPORTED comic showing our ‘multiple tables’ of public involvement (copyright Lilly Williams)

We developed and implemented an ambitious governance framework for public involvement in the study and learned that by including workforce representatives alongside service users and carers, we strengthened important aspects of our work, including the co-design process, the creation and refinement of training resources, and the dissemination of findings and outputs. The quality and diversity of insights shared by public members were more important than the number of public involvement groups, but without that structure, relationships and confidence to speak up would not have developed, enabling learning to take place at multiple levels and acting as a springboard for personal and professional development among public contributors and researchers. Our ‘multiple tables’, with places set for many different people (see Fig. 4 and [11]), meant that we could adapt to challenges without this undermining the process and no one person could either dominate the conversation or damage group dynamics by not engaging. The involvement of people with experience of the workforce under study as public contributors proved to be particularly crucial to the success of the study. Without them, we would (i) not have recruited, (ii) not have gained ethical approval in a timely manner, (iii) not have had the robust insight into the validity and meaning of our findings, and (iv) not have achieved the same level of impact.

## Conclusion

We involved a diverse range of people in research on a sensitive topic in a meaningful way over a sustained period which materially benefitted the study recruitment success and outcomes. Our different experiences wove together to create better, richer research informed by real people and their life experiences. Involving those with experience of the workforce under study proved to be particularly important in achieving our research aims. The experience was both rewarding and challenging. Not everyone will stay engaged, and methods used must be adapted and the people involved added to or changed as issues arise. This is complex and time-consuming, benefiting from someone with a dedicated role to coordinate the work, build relationships and sustain an ongoing dialogue. Equally important are sufficient funding, effective administrative support, and support from research leaders committed to public involvement.

## Data Availability

Access to our data can be made available on request by authorised researchers following completion of a data sharing agreement. To request access, contact the study authors, or (mailto: worktribe@hull.ac.uk) citing Worktribe Output ID 5179695.
